# Cerebral net exchange of large neutral amino acids after lipopolysaccharide infusion in healthy humans

**DOI:** 10.1186/cc8873

**Published:** 2010-02-11

**Authors:** Ronan MG Berg, Sarah Taudorf, Damian M Bailey, Carsten Lundby, Fin Stolze Larsen, Bente Klarlund Pedersen, Kirsten Møller

**Affiliations:** 1 Centre of Inflammation and Metabolism, Department of Infectious Diseases, Rigshospitalet, Blegdamsvej 9, 2100 Copenhagen, Denmark; 2 Neurovascular Research Laboratory, Faculty of Health, Science and Sport, University of Glamorgan, Pontypridd, South Wales CF37 1DL, UK; 3 Copenhagen Muscle Research Centre, Rigshospitalet, Blegdamsvej 9, 2100 Copenhagen, Denmark; 4 Department of Hepatology, Rigshospitalet, Blegdamsvej 9, 2100 Copenhagen, Denmark; 5 Department of Cardiothoracic Anaesthesia and Intensive Care Unit 4131, Rigshospitalet, Blegdamsvej 9, 2100 Copenhagen, Denmark

## Abstract

**Introduction:**

Alterations in circulating large neutral amino acids (LNAAs), leading to a decrease in the plasma ratio between branched-chain and aromatic amino acids (BCAA/AAA ratio), may be involved in sepsis-associated encephalopathy. We hypothesised that a decrease in the BCAA/AAA ratio occurs along with a net cerebral influx of the neurotoxic AAA phenylalanine in a human experimental model of systemic inflammation.

**Methods:**

The BCAA/AAA ratio, the cerebral delivery, and net exchange of LNAAs and ammonia were measured before and 1 hour after a 4-hour intravenous infusion of *Escherichia coli *lipopolysaccharide (LPS) in 12 healthy young men.

**Results:**

LPS induced systemic inflammation, reduced the BCAA/AAA ratio, increased the cerebral delivery and unidirectional influx of phenylalanine, and abolished the net cerebral influx of the BCAAs leucine and isoleucine. Furthermore, a net cerebral efflux of glutamine, which was independent of the cerebral net exchange of ammonia, was present after LPS infusion.

**Conclusions:**

Systemic inflammation may affect brain function by reducing the BCAA/AAA ratio, thereby changing the cerebral net exchange of LNAAs.

## Introduction

Sepsis-associated encephalopathy (SAE) is often one of the first manifestations of sepsis [1] and is associated with an adverse outcome [2,3]. The pathogenesis of SAE is largely unknown, although several potential mechanisms have been investigated, including cerebral blood flow (CBF) and metabolic alterations, intracranial hypertension, cerebral edema, disruption of the blood-brain barrier (BBB), neuronal degeneration, and abnormal neurotransmitter composition [4].

Sepsis is characterized by increased peripheral protein breakdown, notably in skeletal muscle [5,6], and hepatic synthesis of acute-phase reactants; the ensuing alterations in plasma amino acids may play a key role in SAE. Thus, the plasma ratio between branched-chain and aromatic amino acids (BCAAs and AAAs, respectively) decreases, because the BCAAs are rapidly used in the liver, whereas phenylalanine levels increase [7-9]. BCAAs and AAAs belong to the group of large neutral amino acids (LNAAs), which compete for the same saturable carrier across the BBB [10]. Hence, a decrease in the BCAA/AAA ratio theoretically implies either a decreased availability of BCAAs to the brain, or an intracerebral accumulation of AAAs, both of which may profoundly affect neuronal function [11].

At present, neither the physiological implications of alterations in the BCAA/AAA ratio nor the effects of systemic inflammation on the cerebral net exchange of LNAAs has been investigated in humans. Applying a human experimental model of systemic inflammation, we hypothesised that the BCAA/AAA ratio decreases with a concurrent net cerebral influx of the neurotoxic AAA phenylalanine, and that this attenuates the net cerebral influx of BCAAs.

## Materials and methods

Twelve healthy male volunteers aged 20 to 33 (median, 26) years participated in the study after providing oral and written informed consent. All had an unremarkable medical history, with no signs of infection within 4 weeks ahead of the trial day, and none took regular medication. Before inclusion, volunteers underwent a thorough physical examination; a 12-lead electrocardiogram (ECG) was obtained, and standard biochemical tests were performed; all tests were normal. The study was approved by the Scientific Ethical Committee of Copenhagen and Frederiksberg Municipalities, Denmark (file number (KF) 01 290011) and was performed in accordance with the Helsinki Declaration.

Volunteers reported to the laboratory at 7.00 a.m. after an overnight fast and were placed in bed. They were subsequently catheterized with antecubital catheters bilaterally (for saline and lipopolysaccharide (LPS), respectively), a peripheral arterial line and a jugular bulb catheter, of which the two latter were inserted by using local anesthesia with lidocaine. The jugular bulb catheter was inserted into the right internal jugular vein with the tip pointing cranially and by using ultrasound guidance. Correct placement in the jugular bulb was ascertained by feeling a resistance to further advancement of the catheter, as well as the volunteer hearing a purl during a bolus injection of saline; x-ray confirmation was not used. One of the authors (KM) inserted all catheters, including the jugular bulb catheters. After catheter insertion, the volunteer rested in the supine position with slight head elevation for 30 minutes before measurements. Heart rate (via a three-lead ECG), blood pressure, and capillary oxygen saturation were continuously monitored. Volunteers were discharged from the unit after 12 hours after removal of catheters and a light meal. No complications occurred.

### Study design

After an overnight fast, subjects were catheterized, and CBF measurements and paired arterio-jugular venous blood samples were obtained at baseline and after a 4-hour continuous intravenous infusion of purified *Escherichia coli *LPS (infusion rate, 0.075 ng/kg/h; total dose, 0.3 ng/kg); Batch G2 B274, US Pharmacopeial Convention, Rockville, MD, USA). In this model, plasma tumor necrosis factor (TNF)-α peaks at approximately 1 hour after cessation of infusion [12], at which time the second CBF measurement was performed. CBF was determined by means of the Kety-Schmidt technique, as described elsewhere [13].

### Blood gases, pH, haemoglobin, and glucose

Arterial and jugular oxygen tension (PO_2_), carbon dioxide tension (PCO_2_), pH, plasma glucose, and hemoglobin were determined on a blood-gas analyser (ABL 605, Radiometer, Brønshøj, Denmark). For the subsequent calculation of cerebral metabolic rates (CMRs), arterial and jugular venous PO_2 _and plasma glucose were converted to whole-blood oxygen and glucose content, respectively. The oxygen content (C_x_O_2_) in arterial and jugular venous whole blood was calculated as

in which SO_2 _is the oxygen-saturation fraction and Hgb is the concentration of hemoglobin. Whole-blood glucose was calculated as

### Amino acids

Because the plasma and whole-blood concentrations for large neutral amino acids are the same in humans [14,15] and because separation of amino acids is difficult in whole blood, concentrations were determined in plasma, although the exchange between brain and blood of amino acids takes place from both plasma and red blood cells [14,16].

Paired blood samples were simultaneously drawn from the radial artery and the jugular vein after each CBF measurement. The blood was immediately transferred to chilled, heparinized glass tubes, placed on ice, and allowed to equilibrate for 10 minutes. After this, they were centrifuged at 4°C, 3,600 rpm, for 15 minutes. The resultant heparinized plasma was precipitated with sulfosalicylic acid (6%) containing the internal standard for the analysis, norleucine. Samples were then placed on ice for 15 minutes, after which they were centrifuged at 4°C, 3,000 rpm for 30 minutes. With this approach, the amino acid concentration of plasma and red blood cells is fully equilibrated, so that the plasma concentration of a given large neutral amino acid can be considered identical to the whole-blood concentration [17]. Accordingly, the plasma concentrations of amino acids at baseline, measured in the present study, were comparable to whole-blood concentrations of amino acids in humans reported previously [17]. The supernatant was frozen and stored at -80°C until analysis.

LNAAs (phenylalanine, tryptophan, tyrosine, valine, leucine, isoleucine, methionine, histidine, threonine, and glutamine) were separated by a single-column gradient lithium cation-exchange high-performance liquid chromatography with fluorescence detection (Waters HPLC system, Milford, MA), by using post-column derivatization with *o*-phthalaldehyde-mercaptoethanol [18]. The coefficient of variation for all amino acid measurements is less than 5% in this setup.

### Ammonia

To explicate whether a putative cerebral glutamine efflux depended on a cerebral influx of ammonia, the cerebral delivery and net exchange of ammonia were evaluated. The term "ammonia" is used here to depict the total of the charged (NH_4_^+^) and uncharged (NH_3_) species.

Immediately after each CBF measurement, paired blood samples from the radial artery and the jugular vein were simultaneously drawn into EDTA tubes and placed on ice. Within 30 minutes, they were centrifuged at 4°C, 3,600 rpm for 15 minutes; plasma was stored at -80°C. The plasma concentration of ammonia was determined by use of microdiffusion, quantitation by reaction with bromophenol blue, and spectrophotometry at 600 nm (Kodak Ektachem 700 Analyzer, Clinical Chemistry Slide; Eastman Kodak Co., Rochester, NY, USA) at the Department of Clinical Biochemistry, Rigshospitalet. In this assay, NH_4_^+^, which represents more than 98% of ammonia in blood under normal physiologic conditions [19], is directly measured. Any NH_3 _in the sample is converted to NH_4_^+^ during the course of the assay; in effect, total ammonia (NH_4_^+^ + NH_3_) is quantitated. The range of the assay is 6 to 587 μmol/L, and internal validation revealed a coefficient of variation of 5% for values greater than 60 μmol/L and of 11% for values less than this. The whole-blood ammonia concentration was calculated by Conn's formula [20]:

and converted from μg per 100 mL into μmol/L.

### Markers of inflammation

White blood cell and platelet counts were analyzed with standard laboratory methods. The plasma concentration of TNF-α was measured by using ELISA (R&D Systems, Minneapolis, MN, USA). Plasma was obtained by centrifuging whole blood in EDTA-containing tubes at 3,600 rpm at 4°C for 15 minutes and was kept at -80°C until analysis. Samples were analyzed in duplicate, and mean concentrations were calculated. Interassay variability (CV) was assessed by using two internal controls (human plasma); one in the lower end of the standard curve ("low," range 0.86 to 1.35 ng/L), and one in the upper end of the standard curve ("high", range 4.32 to 5.26 ng/L), as the variability differs throughout the standard curve. Interassay CVs were 32.9% for "low" and 12% for "high" TNF-α. The TNF-α detection limit is 0.12 ng/L, according to the manufacturer.

### Calculations

The BCAA/AAA ratio was calculated as the ratio between the arterial plasma concentrations of the BCAAs valine, leucine and isoleucine and the AAAs phenylalanine and tyrosine[21]:

The cerebral delivery of a given LNAA was calculated as the product of the arterial concentration and CBF. Cerebral net exchange (unidirectional cerebral influx - unidirectional cerebral efflux) values of LNAAs and the CMR of oxygen (CMRO_2_) and glucose (CMR_glc_) were calculated according to the Fick principle [22]:

in which J_x _designates the net flux (that is, the cerebral net exchange or CMR) of a given substance x; a-jvD_x _depicts the arterio-jugular venous concentration difference of x in whole blood. By convention, a positive value of J_x _signifies a net influx (uptake) of x, whereas a negative value indicates a net efflux (release) [22].

Given that the BBB transport of a substance can be described accurately by means of a single-membrane model in which the cerebrovascular endothelium behaves as a single membrane, which is the case for LNAAs [23], the unidirectional cerebral influx of phenylalanine (J_in, Phe_) can be calculated

where PS_1 _is the permeability-surface area product of phenylalanine from the capillary into the brain interstitial space. Because the kinetic constants for the transfer of phenylalanine from blood to brain have been estimated in humans *in vivo *[24], and have been found to be similar to values obtained by direct measurements on human brain capillaries after death [25], PS_1 _can be estimated by means of the Michaelis-Menten equation:

In the present context, the maximum transport velocity, V_max_, was assumed to be 46.7 nmol/g/min, the apparent Michaelis-Menten constant, *K*_m_, was assumed to be 0.328 mmol/L, and *K*_D_, the nonsaturable diffusion constant, was assumed to be 0.01 ml/g/min [24].

### Statistics

Parametric methods were applied throughout by using SAS statistical software, version 9.1. Thus, all analyses were performed as paired-samples *t *tests, before and after LPS infusion, to detect an effect of the intervention, and between arterial and jugular venous concentrations at a given time point to determine whether a calculated cerebral net exchange value was different from 0, that is, whether a cerebral influx or efflux was present. Data are presented as mean (95% CI) or as geometric mean (95% CI) in case data had to be log-transformed to achieve normality. Significance was established at *P *< 0.05.

## Results

LPS infusion was associated with a pronounced inflammatory response; immunologic variables are summarized in Table 1. CBF remained unchanged (baseline, 77 (55-101) mL/100 g/min; LPS, 79 (56-109) mL/100 g/min; NS). A mild hyperventilatory response with a decrease in arterial PCO_2 _(baseline, 44.0 (42.5-45.5) mmHg; LPS, 38.8 (36.0-41.6) mmHg; *P *< 0.01), and an increase in pH (baseline, 7.39 (7.38-7.40); LPS, 7.42 (7.39-7.44); *P *< 0.05) was evident after LPS infusion. CMRO_2 _increased slightly (baseline, 1.9 (1.7-2.2) μmol/g/min; LPS, 2.3 (2.0 - 2.6) μmol/g/min; *P *< 0.05), whereas CMR_glc _was unaffected (baseline, 0.36 (0.31-0.40] μmol/g/min; LPS, 0.39 (0.34-0.44) μmol/g/min; NS). Some volunteers dozed intermittently but remained easily rousable and alert when awakened and were fully awake during measurements; no overt signs of encephalopathy occurred.

**Table 1:** Markers of inflammation and large neutral amino acids

	Baseline	LPS
**Temperature (°C)**	36.3 (36.0-36.6)	38.0 (37.6-38.6)‡‡
**Total white blood cells (10**^**9**^**/L)**	5.0 (4.5-5.5)	9.0 (8.0-10.2)‡‡
**Neutrophils (10**^**9**^**/L)**	2.7 (2.3-3.1)	7.6 (6.6-8.7)‡‡
**Lymphocytes (10**^**9**^**/L)**	1.5 (1.3-1.8)	0.8 (0.6-1.0)‡‡
**TNF-α (ng/L)**	0.9 (0.5-1.4)	10.4 (8.7-12.4)‡‡

**BCAA/AAA ratio**	5.2 (4.7-5.7)	4.9 (4.4-5.3)††

**Phenylalanine (μmol/L)**	36 (32-41)	41 (38-44)††
**Tryptophan (μmol/L)**	34 (31-38)	28 (25-32)††
**Tyrosine (μmol/L)**	41 (35-47)	34 (31-39)†
**Valine (μmol/L)**	224 (201-247)	200 (185-214)††
**Leucine (μmol/L)**	113 (101-26)	104 (95-112)†
**Isoleucine (μmol/L)**	59 (53-60)	57 (54-62)
**Methionine (μmol/L)**	14 (13-15)	9 (8-10)‡‡
**Histidine (μmol/L)**	74 (70-78)	58 (52-63)‡‡
**Threonine (μmol/L)**	102 (95-109)	74 (66-82)‡‡
**Glutamine (μmol/L)**	613 (556-669)	449 (411-486)‡‡

AAA = aromatic amino acid; BCAA = branched-chain amino acid, LPS = lipopolysaccharide; TNF-α: tumor necrosis factor α. Different from baseline: †*P *< 0.05; ††*P *< 0.01; ‡‡*P *< 0.0001.

**Figure 1:**
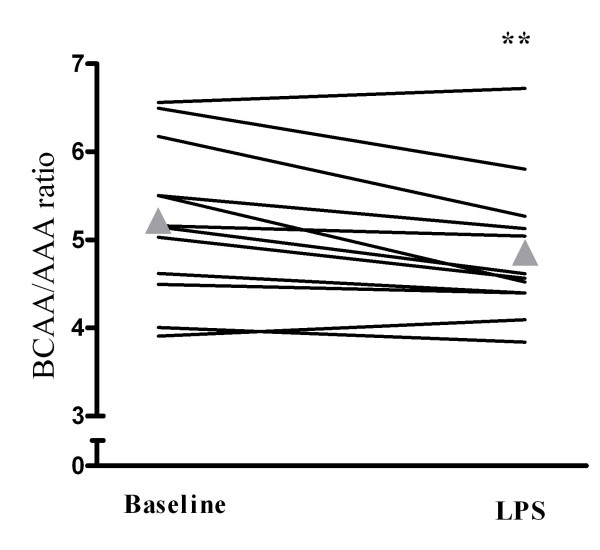
**Branched-chain to aromatic amino acid (BCAA/AAA) ratio after lipopolysaccharide (LPS) infusion in healthy humans**. Triangles indicate means. **Different from baseline, *P *< 0.01.

**Figure 2:**
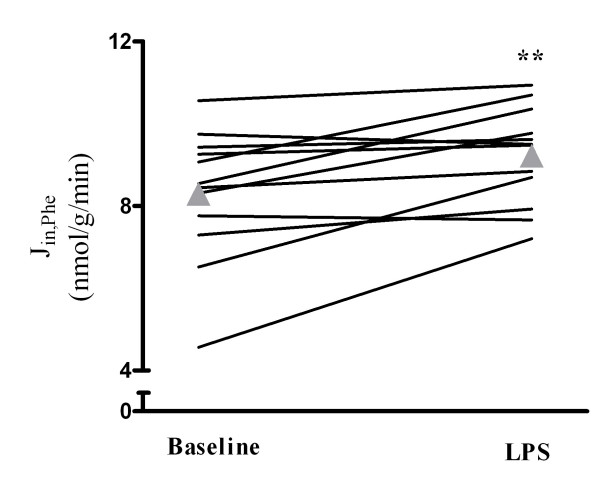
**Unidirectional cerebral influx of phenylalanine (J_in, Phe_) after lipopolysaccharide (LPS) infusion in healthy humans**. Triangles indicate means. **Different from baseline, *P *< 0.01.

LPS infusion increased plasma phenylalanine and decreased the concentration of all other LNAAs except isoleucine (Table 1), with a concurrent reduction in the BCAA/AAA ratio (baseline, 5.2 (4.7-5.7); LPS, 4.9 (4.4-5.3); Figure 1). Both the cerebral delivery (Table 2) and the unidirectional cerebral influx of phenylalanine increased (baseline, 8.3 (6.7-9.9) nmol/g/min; LPS, 9.2 (8.9-10.4) nmol/g/min; Figure 2), whereas its cerebral net exchange was unchanged (Table 3). Furthermore, a net cerebral influx observed at baseline for leucine and isoleucine was abolished after LPS infusion (Table 3). At baseline, a net cerebral influx of methionine was present; this was converted to a net cerebral efflux after LPS infusion (Table 3). Furthermore, a net cerebral efflux of glutamine that was not observed at baseline was present after LPS infusion (Table 3). There was no effect of LPS infusion on the arterial whole-blood concentration of ammonia (baseline, 78 (72-84] μmol/L; LPS, 69 (61-76) μmol/L; NS). The cerebral net exchange of ammonia did not differ from 0 at any time and was unaffected by LPS infusion (baseline, 139 ([-70] - 348) nmol/100 g/min; LPS, -98 ([-618] - 422) nmol/100 g/min; NS).

**Table 2:** Cerebral delivery of large neutral amino acids

Amino acid	Cerebral delivery μmol/100 g/min Baseline	Cerebral delivery μmol/100 g/min LPS
**Phenylalanine**	2.8 (2.4-3.2)	3.3 (2.9-3.6)†
**Tryptophan**	2.6 (2.3-3.0)	2.3 (1.9-2.8)
**Tyrosine**	3.1 (2.7-3.7)	2.8 (2.3-3.2)
**Valine**	17.2 (14.3-20.1)	16.2 (13.7-18.6)
**Leucine**	8.8 (7.1-10.4)	8.4 (7.1-9.6)
**Isoleucine**	4.6 (3.8-5.3)	4.7 (4.0-5.4)
**Methionine**	1.1 (1.0-1.2)	0.7 (0.6-0.9)‡†
**Histidine**	5.7 (4.9-6.5)	4.6 (4.0-5.3)†
**Threonine**	7.7 (6.5-9.0)	5.8 (4.9-6.9)††
**Glutamine**	47.3 (39.6-55.1)	36.3 (30.7-42.0)††

LPS = lipopolysaccharide. Different from baseline, †*P *< 0.05; ††*P *< 0.01; ‡†*P *<0.001.

**Table 3:** Cerebral net exchange of large neutral amino acids

Amino acid	Cerebral net exchange nmol/100 g/min Baseline	Cerebral net exchange nmol/100 g/min LPS
Phenylalanine	22 ([-123]-168)	-17 ([-243]-210)
Tryptophan	-62 ([-251]-125)	-166 ([-408]-77)
Tyrosine	197 ([-67]-461)	-326 ([-665]-14)
Valine	788 (116-1,460)	-331 ([-1,158]-496)
Leucine	650 (263-1,037)**	159 ([-253]-571)
Isoleucine	344 (147-541)*	70 ([-161]-300)
Methionine	64 (8-12)*	-42.9 ([-76]- [-9])††*
Histidine	126 ([-162]-415)	-215 ([-502]-72)
Threonine	164 ([-157]-485]	-355 ([-765]-56)
Glutamine	-305 ([-241]-1,805)	-3651 ([-6,038]- [-1,260])**

LPS = lipopolysaccharide. Different from baseline, ††*P *< 0.01. Cerebral net exchange different from 0, *P < 0.05; **P < 0.01.

## Discussion

The present study is the first to investigate the physiological impact of a decline in the BCAA/AAA ratio after a standardized systemic inflammatory stimulus in humans. In this study, systemic inflammation with an increase in temperature, total white blood cell count, neutrophil count, and plasma TNF-α was associated with a decline in the BCAA/AAA ratio, mainly because of an increase in the arterial concentration of the neurotoxic AAA phenylalanine. A concordant increase in the cerebral delivery and unidirectional cerebral influx of phenylalanine was present during systemic inflammation; this was accompanied by an abolished net cerebral influx of the BCAAs leucine and isoleucine, as well as a cerebral efflux of glutamine. Assuming that the measured cerebral net exchange values are representative for the entire period after LPS infusion, the present findings furthermore indicate that the brain does not contribute to the depletion of glutamine and BCAAs from the circulation during systemic inflammation.

A decrease in the BCAA/AAA ratio was previously demonstrated in patients with sepsis, and this appears to be related to the occurrence of encephalopathy [7-9]. Although the cerebral oxidative metabolism was largely intact, and, as expected, no overt signs of SAE occurred in the subjects, the present human experimental model of sepsis may clarify some underlying concept in the cerebral pathophysiology of sepsis. A decline in the BCAA/AAA ratio was evident after LPS infusion, and this was associated with a remarkable increase in the estimated unidirectional influx of phenylalanine. Thus, if it is assumed that the kinetic constants for phenylalanine transfer across the BBB are not affected by the LPS challenge, the present study provides direct evidence that links the alterations in the BCAA/AAA ratio with changes in the brain's amino acid content in the context of systemic inflammation. Because we did not detect any changes in the cerebral net exchange of phenylalanine, our findings furthermore imply that a new steady state, with elevated phenylalanine levels in the cerebral interstitial fluid, had been established before the second measurement [23]. Previous studies demonstrated increased cerebrospinal fluid (assumed to be representative of the cerebral interstitial fluid) levels of phenylalanine in patients with sepsis [26], which likely affects central noradrenergic pathways by commencing the generation of "false" neurotransmitters, such as phenylethanolamine [11,27]. An unchanged cerebral net exchange could furthermore involve changes in BBB function *per se *through a compensatory increase in the unidirectional cerebral efflux of phenylalanine, a parameter that was not assessed in the present study. This could involve the energy-dependent LNAA transporters on the abluminal membrane of the BBB, which exports phenylalanine from the brain [28,29].

Because AAAs and BCAAs compete for transport across the BBB into the brain by means of the same saturable LNAA carrier [10], the increased arterial phenylalanine levels may furthermore affect cerebral function by reducing the availability of BCAAs to the brain. Concordant with this notion, we found that the observed BCAA/AAA ratio decrease was associated with an abolished cerebral influx of the BCAAs leucine and isoleucine. Of these two BCAAs, leucine is particularly important in the brain, in which it serves as an amino donor for glutamate synthesis in neurons, thus ensuring sufficiently high intracellular concentrations of glutamate for neuronal glutamatergic signaling [30]; in effect, an abolished cerebral influx of leucine may impair excitatory neurotransmission. Although the available data from previous studies are not unequivocal [31-33], it was previously reported that restoration of the BCAA/AAA ratio, by means of treatment with BCAA-rich solutions, decreased the intracerebral levels of phenylalanine, reinstated neurotransmitter profiles, and improved symptoms of encephalopathy in clinical and experimental studies of sepsis [8,27,34]. The present findings may thus corroborate conceptually important aspects of the cerebral pathophysiology of sepsis in a human-experimental setup, in which inflammation-induced alterations in the BCAA/AAA ratio are accompanied by alterations in the transcerebral exchange kinetics of LNAAs. Conversely, the observed changes could be caused by inflammation-induced alterations in BBB function.

In the present study, LPS infusion was found to instigate a decrease in the arterial glutamine levels, and a cerebral glutamine efflux accompanied this. The former has repeatedly been demonstrated, both in clinical and experimental studies of sepsis [5,6,35,36]; the latter has been described in patients with fulminant hepatic failure [17], but has not previously been documented in sepsis. This cerebral efflux likely reflects elevated cerebrospinal fluid glutamine, which has been described in patients with SAE [37].

The cerebral glutamine efflux after LPS infusion was not found to be associated with any changes in the cerebral net exchange of ammonia, which is normally detoxified to glutamine. The classic conception that ammonia merely diffuses across the BBB in its uncharged form (NH_3_) was recently disputed [38,39]; although still controversial, compelling evidence suggests that NH_4_^+^, the most abundant form of ammonia in the circulation, is indeed transported across the BBB by means of a specific carrier [19,38]. Neither the arterial levels nor the cerebral delivery of ammonia, the total of NH_4_^+^ and NH_3_, was affected in the present model. Consistent with our findings, both circulating and brain levels of ammonia have been reported to be unaffected by LPS infusion in rats [40]. Hyperammonemia has, nevertheless, been demonstrated in some animal models of sepsis [40-42] and may aggravate intracranial hypertension in septic rats [43]. Furthermore, the cerebral net exchange values reported in the present study may be prone to inaccuracy, because of the considerable coefficient of variation of the ammonia measurements in the lower range (11%). Combined with the relatively low ambient ammonia concentrations, it is possible that any minor arterio-jugular venous concentration differences, which could prompt significant alterations in cerebral net exchange because of the inherently high CBF values, were not detected in our setup. Therefore, we cannot conclusively state that no changes in the cerebral net exchange of ammonia were induced by LPS infusion. In consequence, our findings do not necessarily exclude a pivotal role of ammonia in the pathophysiology of SAE. They do, however, suggest that any putative part played by ammonia in this respect is more likely that of a contributing than of a causative factor, and that a cerebral ammonia uptake is not the solitary cause of the evident cerebral glutamine efflux.

Rather than ammonia detoxification, the cerebral efflux of glutamine after LPS infusion probably reflects increased cerebral proteolysis or a compensatory astrocytic glutamine release, for example, to reduce osmotic stress in the context of cytotoxic edema. As with phenylalanine, an alternative explanation could be the presence of an inflammation-induced increase in the activity in the energy-dependent abluminal LNAA transporters [28], phenomena that are not mutually exclusive. Glutamine supplementation was recently shown to oppose the progressive decline in circulating glutamine levels [35] and to attenuate organ damage in experimental sepsis [36]; however, the impact of glutamine supplementation on brain function and symptoms of encephalopathy in sepsis remains to be elucidated.

Certain limitations exist for the conclusions that can be made from our findings. Based on the methods and findings in the present study, we cannot definitively conclude that the cerebral net exchange of a given amino acid is unaffected in sepsis. The cerebral net exchange values are relatively small at baseline (Table 3), and the clinical impact of LPS infusion is much less than that of full-scale sepsis, although the two scenarios are similar with regard to the cytokine response [44]. It is, therefore, possible that the immune response triggered in this model is not sufficient to cause alterations in the cerebral net exchange of at least some LNAAs of a magnitude that can be detected by the methods applied; the signal-to-noise ratio may be too low, and the duration of systemic inflammation needed for the development of such changes may be longer than that evoked by a 4-hour LPS infusion. In addition, the Kety-Schmidt technique exclusively assesses global CBF and metabolism; consequently, potential regional changes remain unveiled. Such changes may in reality be present both during experimental systemic inflammation and in full-scale sepsis. Nonetheless, an immense inflammatory response with biochemical signs of infection was triggered in the present study, and as recently reviewed, a number of confounding factors associated with both clinical and animal studies of sepsis are circumvented in the present model [44]. Hence, LPS infusion in humans appears to be valid for human *in vivo *studies of certain aspects of the pathophysiology of early sepsis.

We did not perform cognitive tests in the volunteers. Thus, possible interrelations between cerebral function and the described alterations in arterial amino acids could not be assessed. It is quite possible that the general malaise experienced by the subjects might have affected cognitive performance, had it been tested thoroughly. However, because all subjects remained alert and responsive during the course of LPS infusion, major cognitive disturbances were unlikely to be present.

## Conclusions

The present study lends further support to the view that LNAAs, particularly phenylalanine, play a pertinent role in the cerebral pathophysiology of sepsis; the arterial levels, cerebral delivery, and unidirectional cerebral influx of phenylalanine increased, whereas the cerebral influx of leucine and isoleucine were abolished, and a cerebral glutamine efflux was induced by LPS infusion in humans. Future studies should address these interrelations and characterize them further, for example through bedside studies on cerebral net exchange, neurotransmitter profiles, BBB function, and the effects of amino acid supplementation on brain function in patients with sepsis.

## Key messages

• LPS infusion induces a systemic inflammatory response and reduces the arterial levels of most LNAAs in humans.

• The systemic inflammatory response is associated with a decrease in the BCAA/AAA ratio, because a reduction in the arterial levels of the BCAAs valine and isoleucine occurs with a concomitant increase in the arterial levels of the neurotoxic AAA phenylalanine.

• The BCAA/AAA ratio decrease is associated with an increase in the cerebral delivery and unidirectional cerebral influx of phenylalanine, an abolished influx of the BCAAs leucine and isoleucine, and an ammonia-independent cerebral efflux of glutamine.

## Abbreviations

AAA: Aromatic amino acid; BCAA: branched-chain amino acid; CBF: global cerebral blood flow; CMR: cerebral metabolic rate; ECG: electrocardiogram; Hgb: hemoglobin; J: flux; LNAAs: large neutral amino acids; LPS: lipopolysaccharide; NH: ammonia; SAE: sepsis-associated encephalopathy.

## Competing interests

The authors declare that they have no competing interests.

## Authors' contributions

RMGB conducted the study, acquired, analyzed, and interpreted the data, performed statistical analyses, and drafted the manuscript. ST, DMB, and CL conducted the study and acquired and interpreted the data. FSL conducted the amino acid analyses. BKP conceived of and designed the research and handled funding and supervision. KM conceived of and designed the research, conducted the study, acquired, analyzed, and interpreted the data, drafted the manuscript, and handled funding and supervision. All authors made critical revisions and read and approved the final manuscript.
